# Machine learning-based prediction models for parathyroid carcinoma using pre-surgery cognitive function and clinical features

**DOI:** 10.1038/s41598-023-46294-7

**Published:** 2023-11-03

**Authors:** Yuting Wang, Bojun Wei, Teng Zhao, Hong Shen, Xing Liu, Jiacheng Wang, Qian Wang, Rongfang Shen, Dalin Feng

**Affiliations:** Department of Thyroid and Neck Surgery, Beijing Chaoyang Hospital, Capital Medical University, Beijing, China

**Keywords:** Cancer models, Endocrine cancer, Head and neck cancer, Cancer, Endocrinology, Endocrine system and metabolic diseases, Computational biology and bioinformatics, Machine learning, Predictive medicine, Learning and memory

## Abstract

Patients with parathyroid carcinoma (PC) are often diagnosed postoperatively, due to incomplete resection during the initial surgery, resulting in poor outcomes. The aim of our study was to investigate the pre-surgery indicators of PC and try to develop a predictive model for PC utilizing machine learning. Evaluation of pre-surgery neuropsychological function and confirmation of pathology were carried out in 133 patients with primary hyperparathyroidism in Beijing Chaoyang Hospital from December 2019 to January 2023. Patients were randomly divided into a training cohort (n = 93) and a validating cohort (n = 40). Analysis of the clinical dataset, two machine learning including the extreme gradient boosting (XGBoost) and the least absolute shrinkage and selection operator (LASSO) regression were utilized to develop the prediction model for PC. Logistic regression analysis was also conducted for comparison. Significant differences in elevated parathyroid hormone and decreased serum phosphorus in PC compared to (BP). The lower score of MMSE and MOCA was observed in PC and a cutoff of MMSE < 24 was the optimal threshold to stratify PC from BP (area under the curve AUC 0.699 vs 0.625). The predicted probability of PC by machine learning was similar to the observed probability in the test set, whereas the logistic model tended to overpredict the possibility of PC. The XGBoost model attained a higher AUC than the logistic algorithms and LASSO models. (0.835 vs 0.683 vs 0.607). Preoperative cognitive function may be a probable predictor for PC. The cognitive function-based prediction model based on the XGBoost algorithm outperformed LASSO and logistic regression, providing valuable preoperative assistance to surgeons in clinical decision-making for patients suspected PC.

## Introduction

Parathyroid carcinoma (PC) is a rare malignant tumor that accounts for 0.5–5% of patients with primary hyperparathyroidism(PHPT) and only 0.005% of all cancers^1–3^. In European and Asia countries, the mean incidence of PC has increased similarly over time, which might be attributed to the rise in PC diagnoses brought on by the prevalence of parathyroid diseases and the growing rate of PHPT suffering parathyroidectomy^4,5^.

In distinction to local excision of parathyroid adenoma (PA), en bloc resection as a treatment for parathyroid cancer, particularly during the initial surgical treatment has a critical impact on patient prognosis, which emphasizes the significance of preoperative diagnosis^6–8^. However, diagnosing PC before surgery is difficult, mainly because there are no definitive preoperative markers for PC. Due to similar clinical manifestations, PC is often misdiagnosed preoperatively and treated as a benign parathyroid disease (BP). The histological definition of WHO criteria for PC required an infiltrative growth pattern or metastasis^9^. Preoperative fine needle aspiration (FNA) and intraoperative biopsy are insufficient to diagnose if definitive histopathological criteria of invasion is absent in some PC specimens^10,11^. Additionally, patients with FNA increase the risk of tumor cell seeding along the needle tract. The presurgical prediction for PC is still challenging.

An increasing number of PHPT patients present primarily with neuropsychological symptoms, such as cognitive deficits, anxiety, and poor concentration, rather than skeletal and renal complications like osteoporosis and nephrolithiasis resulting from long-term hypercalcemia, highlighting the value of assessing neuropsychological manifestations^12,13^. Moreover, recent studies have demonstrated that patients with non-central nervous system tumors frequently suffer cognitive impairment even before undergoing treatments associated with toxicity, including chemotherapy, immunotherapies, and radiation^14,15^. However, due to the rarity of the disease, only a few studies have evaluated cognitive impairment in PHPT by examining a limited number of cognitive domains^16–18^. Additionally, there is a paucity of research assessing cognitive function specifically in patients with PC, further limiting the investigation of the relevant variables affecting preoperative cognitive decline and hampering comparisons between malignant and benign parathyroid diseases.

Machine learning (ML) is developed from the study of pattern recognition and computational learning to minimize errors between predicted and tested sets. Extreme gradient boosting (XGBoost) can distribute the gradient boosting library and imply ML algorithms under the Gradient Boosting framework. Interestingly, XGBoost has been successfully applied to diagnose and predict the prognosis of cancers, such as lung cancer, hepatocellular cancer, breast cancer, and lung metastases from thyroid cancer^19–22^. The Least absolute shrinkage and selection operator (LASSO) can minimize the residual sum of squares and select the variables most related to the disease than traditional regression^23^. Currently, LASSO has been used for the development of disease prediction and risk model^24–27^.

In the present study, we sought to explore the potential predictive indicators for PC by examining the preoperative cognition functions as well as several serum biomarkers of parathyroid diseases and determining whether they have a relevant association with cognitive function. Besides, this study established prediction models for PC based on the XGBoost algorithm and LASSO regression. To the best of our knowledge, the use of XGBoost and LASSO in the prediction of PC has not ever been reported.

## Methods

### Participants

**Figure 1:**
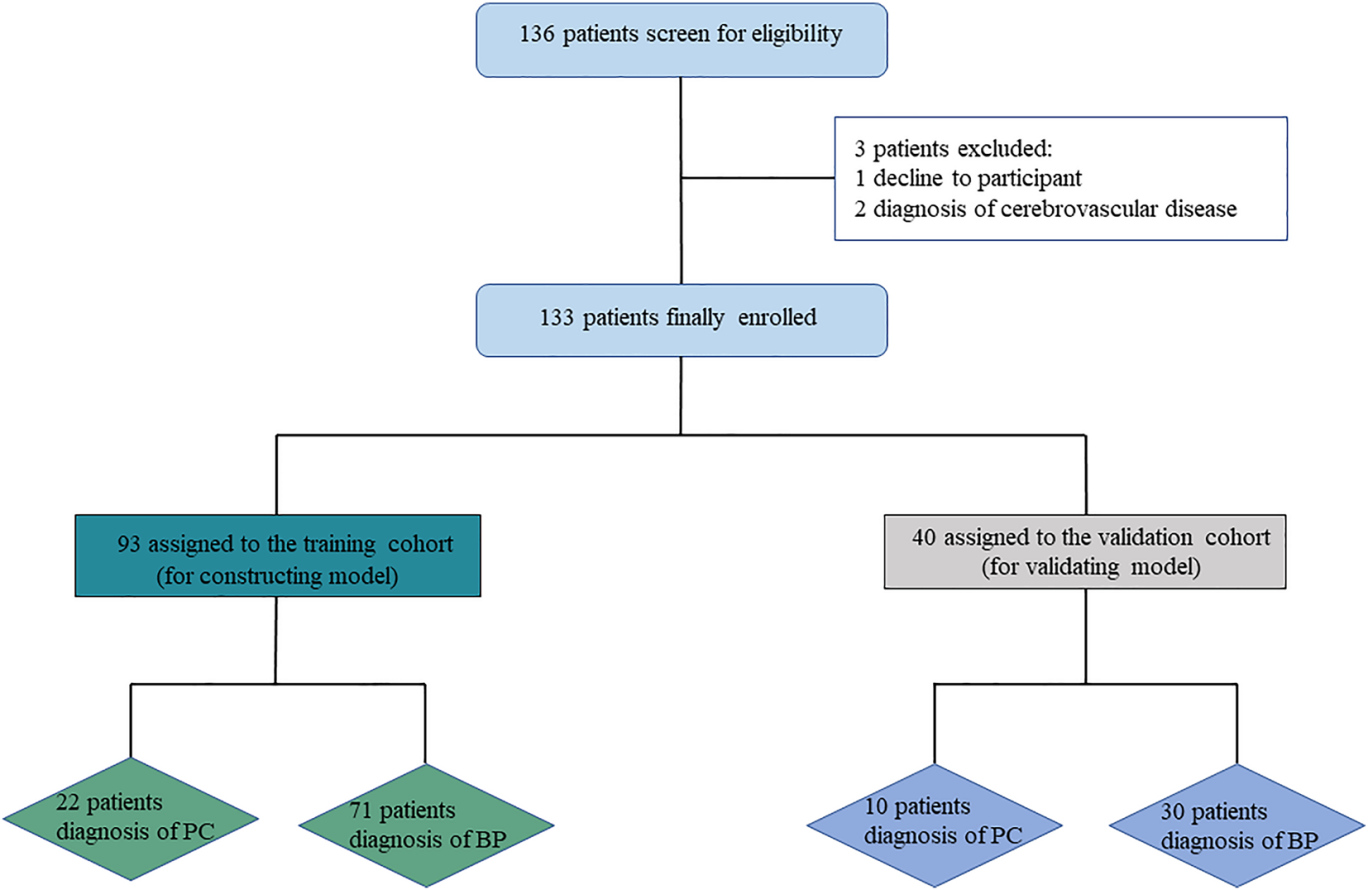
Study design.

This study was approved by the Ethics Committee of Beijing Chaoyang Hospital, China. Informed consent was obtained from all patients participating in this research. All methods were carried out in accordance with the applicable guidelines and regulations. A total of 136 patients were consecutively diagnosed with PHPT based on biochemical criteria (serum calcium > 2.52 mmol/L and PTH > 88 pg/mL) in Beijing Chaoyang Hospital, China, from December 2019 to January 2023. Except for two patients with a history of cerebrovascular disease who were on long-term treatment and one patient who opted for clinical observation instead of surgery, 133 patients performed surgical treatment and were finally enrolled (Fig. 1). The age range of the PHPT patients was between 14 and 70 years. Exclusion criteria include patients with cerebrovascular disorders, dementia, previous head injury, severe cardiovascular diseases, and other malignant neoplasms.

Data of pre-operation clinical features including the history of osteoporosis, fracture, renal stone, and hypercalcemia-related symptoms, laboratory findings (serum level of parathyroid hormone, total calcium, phosphorous, alkaline phosphatase, creatinine, 25-hydroxy vitamin D and 24-h urinary calcium), psychological and neurocognitive function were collected. Between December 2019 and December 2022, 133 PHPT patients were evaluated neuropsychologically within one week before surgery by the same two physicians.

### Diagnostic criteria and follow-up

Pathological diagnoses were made according to WHO criteria by experienced pathology physicians in Beijing Chaoyang Hospital. The BP group consisted of ninety-six patients with parathyroid adenoma (PA), three patients with parathyroid hyperplasia, and two patients with parathyroid cysts, respectively. In our institution, among the PC group, initial En-bloc resection was performed on twenty-three patients (71.88%). The remaining nine PC patients (28.12%) underwent reoperation following an unsuccessful initial operation performed elsewhere. The dataset was randomly split into a training cohort (70%) and a test cohort (30%) without a difference in the baseline (Table 1).

**Table 1:** Baseline characteristics of PHPT patients in the training set and validation set.

Factors	Training set (n = 93)	Validation set (n = 40)	χ^2^	*P*-value
Age (years)	51 ± 1	51 ± 2	0.127	0.808
Sex			0.855	0.355
Male (%)	23 (24.7%)	13 (32.5%)		
Female (%)	70 (75.3%)	27 (67.5%)		
Education (years)	13 ± 0	14 ± 0	− 1.19	0.474
Tumor diameter (cm)	1.70 (1.30, 2.20)	2.00 (1.35, 2.00)		0.998
Creatinine (umol/L)				
Male	74.8 (64.4, 86.8)	71.3 (60.5, 82.8)		0.489
Female	50.5(46.3, 57.2)	50.8 (43.8, 62.2)		0.437
Laboratory tests				
PTH (pg/ml)	151.6 (114.0, 267.3)	197.4 (104.7, 260.3)		0.998
Calcium (mmol/L)	2.64 (2.52, 2.79)	2.71 (2.59, 2.85)		0.148
Phosphorous (mmol/L)	0.88 (0.74, 1.00)	0.85 (0.72, 0.95)		0.359
25-hydroxy vitamin D (ng/ml)	14.18 (11.04, 19.60)	14.96 (11.13, 20.42)		0.783
Alkaline phosphatase (ALP, U/L)	95.5 (69.0, 132.5)	85.0 (68.5, 109.5)		0.294
24H urinary calcium (mmol/24H)	6.60 (4.51, 9.21)	6.88 (5.18, 9.90)		0.260
Neuropsychological evaluation				
MMSE	27 (25, 29)	26 (24, 28)		0.157
MOCA	25 (22, 26)	24 (23, 27)		0.504
HAMD	9 (5, 12)	9 (7, 12)		0.710
HARS	10 (5, 14)	7 (6, 12)		0.524
MAES	2 (1, 4)	2 (1, 4)		0.183
Diagnosis			0.028	0.868
BP	71 (76.3%)	30 (75.0%)		
PC	22 (23.7%)	10 (25.0%)		

PTH: parathyroid hormone Normal range: Calcium: 2.11–2.52 mmol/L, phosphate: 0.85–1.51 mmol/L, PTH: 18.5–88.0 pg/mL, 25(OH)D: 20–100 ng/mL, *p < 0.05 for primary hyperparathyroidism versus benign thyroid disease.

The median follow-up time for the patients was 13.5 months. Among them, 6 patients with PC had distant metastases, with all 6 patients having lung metastases and 2 patients suspected of having bone metastases. None of the patients had definitive recurrence or death.

### Neurocognitive and neuropsychological function assessment

Neurocognitive Assessment. Mini-Mental State Examination (MMSE) was used in objective measures of cognitive function including thirty items categorized into seven groups (Orientation to time and place; Registration; Attention and Calculation; Recall; Language; Visual Construction)^28,29^. The total score is thirty. Mild cognitive impairment (MCI) is defined as a range of 18–24, and severe cognitive impairment as scoring 17 or less. Montreal Cognitive Assessment (MoCA) with a 30-point test including a short-term memory recall task, visuospatial and executive function, language, naming, attention and calculation, abstraction, and orientation was used. To rectify the education impact, 1 point was added for participants with 12 years of education or less on their total score of MOCA (if the score < 30). Scores of 25 or below indicate cognitive impairment^30,31^.

Psychological Instrument. Hamilton Depression Rating Scale (HAMD-17) was used to evaluate depression, which contains 17 elements scored from 0(never) to 4(severe). The severity ranges for the score of HAMD are as follows: no depression (0–7); mild depression (8–16); moderate depression (17–23); and severe depression (≥ 24)^32,33^. Hamilton Anxiety Rating Scale (HARS) was used to assess anxiety. It consists of 14 symptom-defined variables divided into somatic and psychogenic anxiety. Each item is scored from 0 (not present) to 4 (severe): > 8 is considered mild anxiety; 14–56 is considered moderate–severe^34,35^. MAES consisting of 14 items was used to measure the emotional, behavioral, and cognitive aspects of apathy. Questions are rated on a scale from 0(a lot) to 3 (not at all). It defined apathy as having a score of ≥ 14^36–38^.

### Statistical methods

Demographic, clinical, laboratory, histological data, and cognition function were characterized by descriptive statistical methods. As the clinical data of PHPT patients were not normally distributed, the Mann–Whitney U test was used to explore the characteristic variates for their potential of differentiation between the PC and benign groups. The statistical power of the nonparametric test was 0.80, while the alfa error was 0.05, and the sample size was 88 in group 1 and 28 in group 2. The final enrollment in our study was 91 in the benign group and 32 in the malignant group which matched the required sample size. We defined the sex variable as 1 for females and 2 for males. The chi-square test was used between groups (alfa error = 0.05, power = 0.8, minimal sample size = 88). A *p*-value < 0.05 was significant with two-sided. The statistical analyses were conducted using SPSS version 26. 0(IBM Corp. Released 2019. IBM SPSS Statistics for Windows, Version 26.0. Armonk, NY: IBM Corp).

### Model development and model performance evaluation

First, the logistic regression model was used to develop a prediction model including variates with a *p*-value less than 0.05 in the univariate analysis. The anticipated probability of PC computed from the best fitting model was chosen as the prediction criterion. Backward stepwise was conducted to identify significant predictors (*p* < 0.05). The dependent y only takes 0 and 1 as dichotomous variables. ${\text{P}} = {\text{P}}\left( {{\text{y}} = {1}|{\text{x}}_{{1}} , \ldots ,{\text{x}}_{{\text{n}}} } \right)$ is affected by N factors. The formula of P can be obtained like this:

$$ {\text{P}} = \frac{1}{{1 + e^{{ - \left( {\beta 0 + \beta 1x1 + \cdots + \beta nxn} \right)}} }} $$

Secondly, XGBoost is a scalable tree boosting system based on gradient lifting decision trees for classification and regression predictive model, which avoids overfitting by adding regularization terms, using shrinkage scales for added weights, and using column subsampling. this algorithm improves prediction accuracy by working on the principle of optimizing functions. The XGBoost algorithm uses N additive functions to predict output in a tree ensemble model. Each regression tree involves a continuous score on each leaf when T is the number of leaves in the tree and each $f\left(n\right)$ has an independent structure as well as leaf weight. We can measure the difference between the prediction to the target based on this tree. Meanwhile, this model also presents the regression tree^39^.

We also used the LASSO regression to choose the most significant variables. The LASSO regression shrunk the coefficients by imposing a penalty term, named lambda (λ), which is selected by visualization methods and cross-validation. Based on the optimal value of λ, we calculate the coefficients and build the LASSO model^23^.

Receiver operating characteristic (ROC) analysis and measured area under the curve (AUC) were used to compare the efficacy of the predictive model corresponding to pathology. Hosmer–Lemeshow test measured calibration by *p*-value. we evaluated the predictive effect by running this model in the validating cohort. Both the XGBoost algorithm and the LASSO regression were performed with Python version 3.10.

We used the median value of the BP or PC group as supplementation for missing values. There was one PC patient with a missing value of ALP which we supplemented to 109 IU/L and another PC with a deficient value of 24-h urinary calcium which we handled to 5.96 mmol/24H in the training set.

### Ethics approval and consent to participate

This study was approved by the Medical Ethics Committee of Beijing Chao-Yang Hospital, Capital Medical University. Informed consent was obtained from all participants and/or their legal guardians.

## Result

### Clinical characteristics between the training set and the validating set

Among the 93 patients in the training cohort, 23 were male and 70 were female. The mean age of patients was 51 ± 1 years. Of the 40 patients in the validating cohort, 13 were male and 27 were female. The mean age of patients was 51 ± 2 years. There were no differences in sex, age, education, biochemical tests, pre-surgery cognition, psychology, and tumor diameter between the training cohort and the validating cohort (Table 1).

### Comparison of preoperative demography and clinical characteristics between the PC group and BP group

The description of the demography and relevant clinical characteristics were summarized (Table 2).

**Table 2:** Demography and clinical characteristics in patients with PC and BP.

Factors	BP group (n = 101)	PC group (n = 32)	F/χ^2^	*P*-value
Age (years)	51 ± 1	51 ± 2	0.321	0.572
Sex			28.456	0.000*
Male (%)	20 (19.8%)	16 (50.0%)		
Female (%)	81 (80.2%)	16 (50.0%)		
Education(years)	15(11.16)	12(9,16)	20.526	0.153
Presenting symptom				
Asymptomatic (%)	6 (5.9%)	1 (3.1%)	0.222	0.637
Fatigue (%)	52 (51.5%)	16 (50.0%)	0.168	0.682
Palpable mass (%)	3 (3.0%)	3 (9.4%)	0.462	0.497
Dizziness (%)	5 (5.0%)	3 (9.4%)	0.015	0.902
Hoarse (%)	2 (2.0%)	1(3.1%)	1.072	0.301
Bone pain (%)	59 (62.8%)	26 (66.7%)	0.182	0.670
Polydipsia (%)	35 (34.7%)	13 (40.6%)	0.182	0.670
Gastrointestinal-discomfort (%)	32 (31.7%)	11 (34.4%)	0.429	0.512
Arrhythmia (%)	57 (56.4%)	25 (64.1%)	0.140	0.708
Altered mental-status (%)	26 (25.7%)	14 (43.8%)	0.889	0.346
Complications				
Osteoporosis (%)	39 (38.6%)	23 (71.9%)	4.139	0.126
Low bone mineral density (%)	27 (26.7%)	10 (31.3%)	0.516	0.473
Fragility fracture (%)	8 (7.9%)	4 (12.5%)	0	1.000
Spine (%)	1 (1.1%)	1 (3.1%)	0	1.000
Ankle (%)	3 (3.2%)	0	0.237	0.626
Wrist (%)	3 (3.2%)	1 (2.6%)	0	1.000
Humerus (%)	1 (1.0%)	1 (2.6%)	0	1.000
Nephrolith (%)	37 (36.6%)	19 (59.4%)	0.990	0.320
Tumor diameter (cm)	1.60 (1.30, 2.00)	2.00 (1.50, 2.50)		0.066
Creatinine (umol/L)				
Male	67.4 (59.9, 83.5)	76.15 (64.40, 99.00)		0.446
Female	50.5 (45.8, 56.9)	54.40 (47.85, 61.65)		0.218
Laboratory tests				
PTH (pg/ml)	147.7 (111.8, 207.7)	263.1 (117.5, 586.8)		0.004*
Calcium (mmol/L)	2.68 (2.52, 2.83)	2.64 (2.56, 2.83)		0.924
Phosphorous (mmol/L)	0.89 (0.80, 1.00)	0.69 (0.65, 0.81)		0.000*
25-hydroxy vitamin D (ng/ml)	13.83 (10.43, 20.08)	15.57 (13.19, 19.54)		0.204
Alkaline phosphatase (ALP, U/L)	90.0 (69.0, 111.0)	109.0 (69.0, 169.0)		0.057
24H urinary calcium (mmol/24H)	7.00 (4.80, 9.36)	5.96 (4.38, 9.12)		0.502

PTH: parathyroid hormone Normal range: Calcium: 2.11–2.52 mmol/L, phosphate: 0.85–1.51 mmol/L, alkaline phosphatase: 35–100 IU/L, PTH: 18.5–88.0 pg/mL, 25(OH)D: 20–100 ng/mL, creatinine: 57–97 μmol/L♂, 41–81 μmol/L♀, 24H urinary calcium: 2.5–7.5 mmol/24H) *p < 0.05 for parathyroid carcinoma versus benign parathyroid disease.

It was shown that the BP group marked a definite female preponderance. Preoperative PTH was significantly higher and serum phosphorus was lower in those with PC, while no significant difference was noticed between the two groups in other aspects.

**Figure 2:**
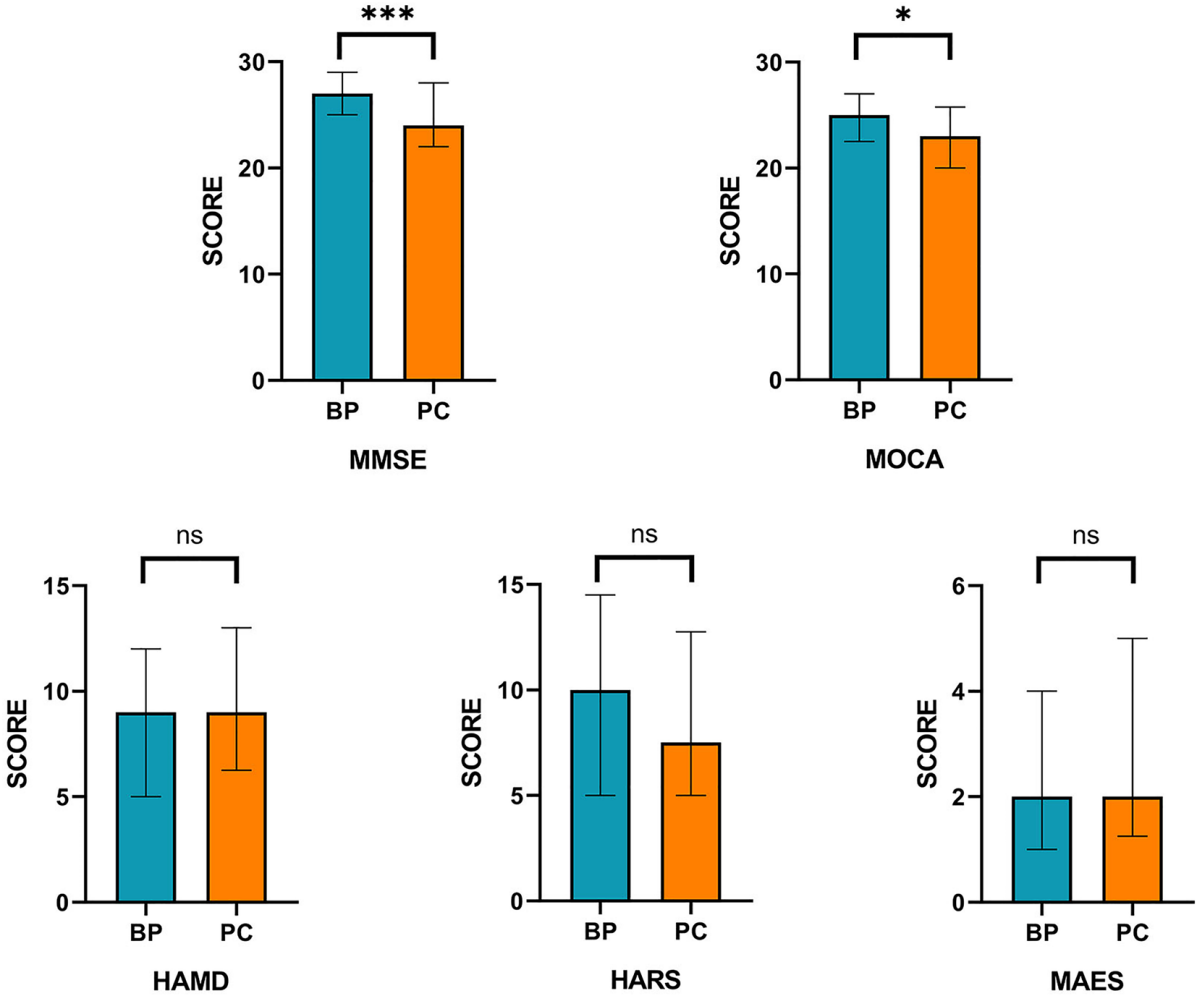
Assessment of cognitive function and psychological changes between PC and BP. ***p < 0.000 for parathyroid carcinoma versus benign parathyroid disease; *p < 0.05 for parathyroid carcinoma versus benign parathyroid disease.

Based on the scores of instruments, neither the PC group nor the BP group reported severe depression, anxiety as well as apathy, and there were no differences in mood (*p* = 0.65, *p* = 0.271, *p* = 0.243). The scores of cognitive function extent to be normal on mean in both PC and BP groups. MMSE and MOCA were significant in discriminating PC from BP (*p* = 0.004, *p* = 0.013), obtained through the Mann–Whitney test. The AUC of MMSE was greater than MOCA (MMSE 0.721vs MOCA 0.646), which confirmed the superiority of MMSE to MoCA in detecting PC. Patients assessed cognitive function and psychological changes, as exposed in Fig. 2.

### Logistic regression analysis of the prediction model for PC

The logistic regression model with backward stepwise is shown in Table 3. The sex of patients was defined as a dummy variable and assigned values at analysis, putting female in 1 and male in 2. Preoperative PTH was positively correlated with the prediction model of PC. In contrast, the score of MMSE and the sex were inversely associated with the prediction model of PC. The final equation developed by the logistic regression model to predict PC was as follows: P = $\frac{1}{1{+e}^{-\left(9.109+0.002\times PTH-0.367\times MMSE-1.847\times sex\right)}}$, the closer the value of *p* is to 1, the higher the probability of parathyroid carcinoma in PHPT.

**Table 3:** Multivariate logistic regression analysis of prediction.

Variable	β	SE	P	OR	95%CI
MMSE	− 0.367	0.113	0.001	0.693	0.555–0.865
PTH	0.002	0.001	0.048	1.002	1.000–1.003
Sex (1)	− 1.847	0.634	0.004	0.158	0.045–0.547

Logistic regression analysis showed that sex (OR 0.158, *p* = 0.004, 95%CI 0.045–0.547), preoperative PTH (OR 1.002, *p* = 0.048, 95%CI 1.000–1.003), and the pre-surgery score of MMSE (OR 0.693, *p* = 0.001, 95%CI 0.555–0.865) might be suggested as possible indicators for PC.

### XGBoost model of the prediction model for PC

**Figure 3:**
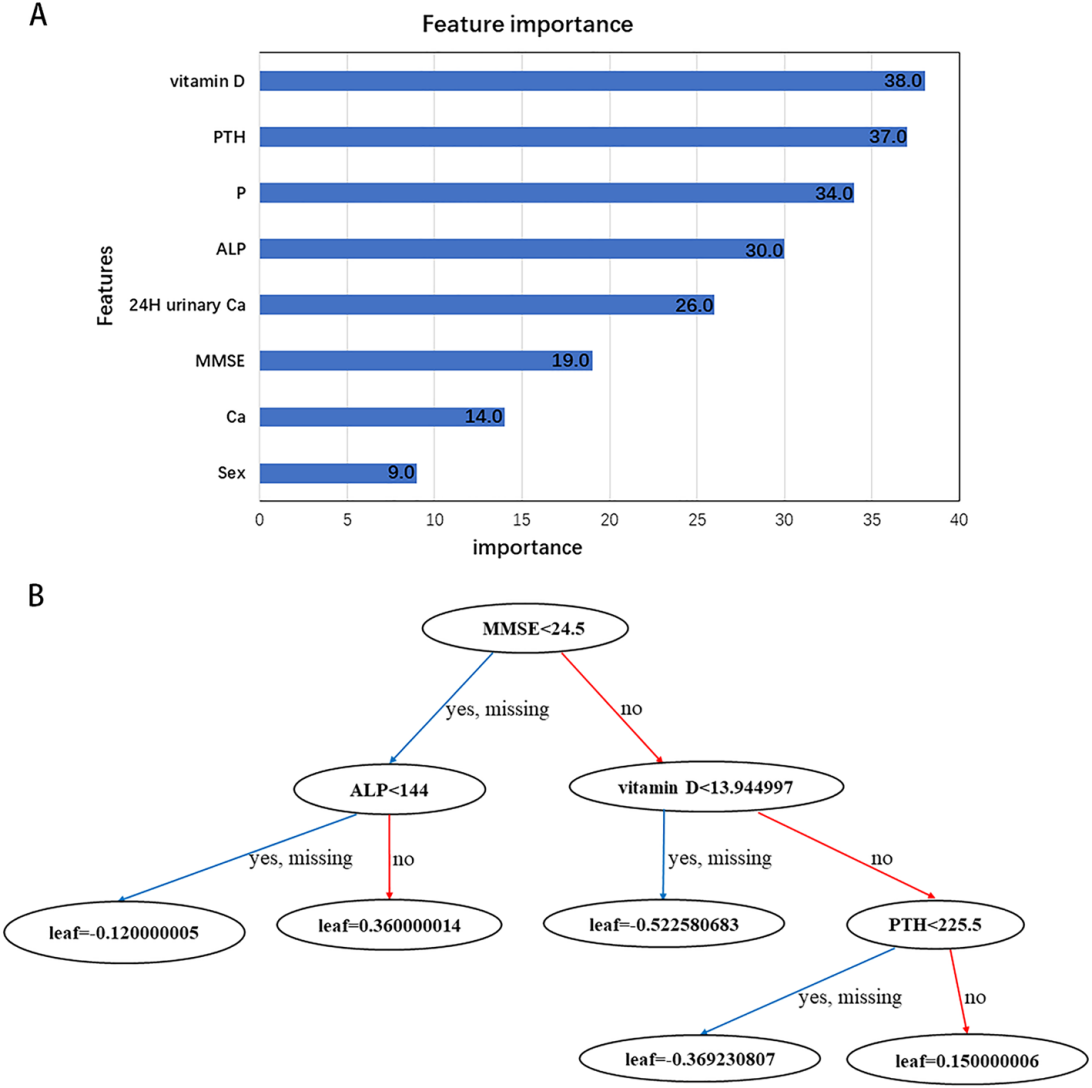
Features importance (**A**) and tree structure of the XGBoost model (**B**). (**A**) Features importance in the training set. PTH: parathyroid hormone. P: serum phosphorous. ALP: alkaline phosphatase. Ca: serum calcium. 24H urinary Ca: 24H urinary calcium. (**B**) The first tree structure of the XGBoost model.

The hyperparameters were selected by cross-validation and grid searches in the XGBoost model, by inputting the sex, all the laboratory test results, and scores of MMSE and MOCA of all the PHPT patients, and the top eight indicators for important features were finally determined by incorporating them into the algorithm model with data of the training set (sex, MMSE, PTH, alkaline phosphatase, calcium, 24-h urinary calcium, 25-hydroxy vitamin D and phosphorous, Scores of important features were shown in Fig. 3A). The value of features in the model for improving decision tree development is used to determine the importance of a feature. An attribute's relative value is increased if it influences split point improvement (the closer it is to the root node) or is chosen by more boosting trees. According to the decision tree structures of the XGBoost model, the predictive values for PC can be calculated and normalized to range from 0–1. The first tree structure was shown in Fig. 3B. if the score of MMSE in patients with PHPT was < 24.5 and the ALP was < 144 (U/L), the probability of PC was $1/[1+\mathrm{exp}(-\mathrm{leaf})$] = $1/[1+exp(-0.360)$] = 0.589.

### LASSO model of the prediction model for PC

**Figure 4:**
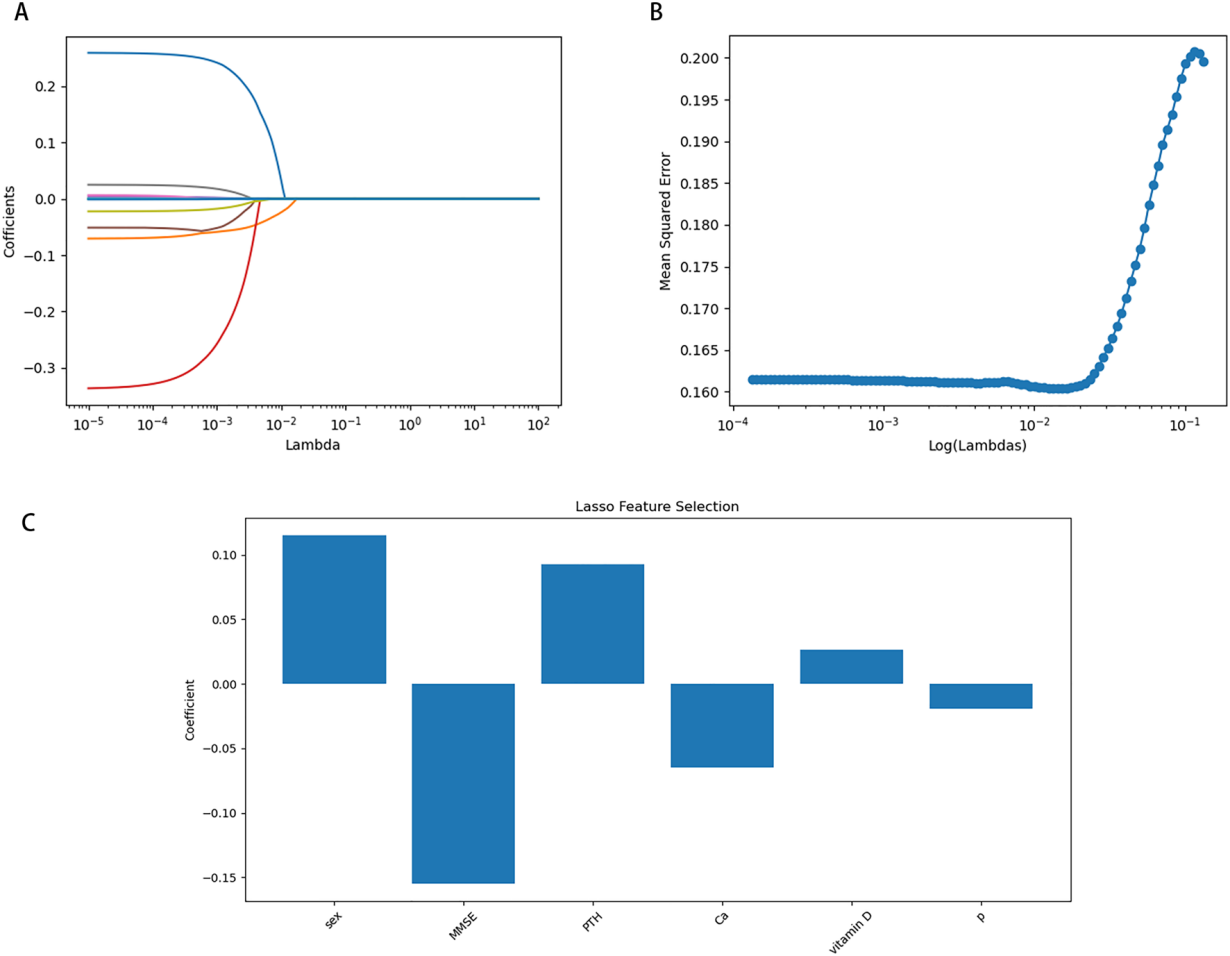
(**A**, **B**, **C**) coefficients, cross-validation, and feature selection in LASSO regression. (**A**, **B**) We ensured optimal penalty parameter lambda (λ) at the minimum mean squared error value. (**C**) The coefficient of 6 variables including sex, MMSE, PTH, calcium, 25-hydroxy vitamin D, and phosphorous, log(λ) = − 2.171463896 (λ = 0.006738079091822886) when the error of the model is minimized, and 6 variables were selected for further regression analysis.

Sex and clinical indicators, including laboratory results and neurocognitive assessment, were subjected to LASSO regression. We utilized cross-validation to ensure the optimal penalty parameter lambda (λ) at the minimum mean squared error value (Fig. 4B). Log(λ) = − 2.171463896 (λ = 0.006738079091822886) minimized the regression coefficient (Fig. 4A) while 6 variables remained in further regression (sex, MMSE, PTH, calcium, 25-hydroxy vitamin D and phosphorous, Fig. 4C).

### Performance in prediction model of PC among XGBoost, LASSO, and logistic regression

**Figure 5:**
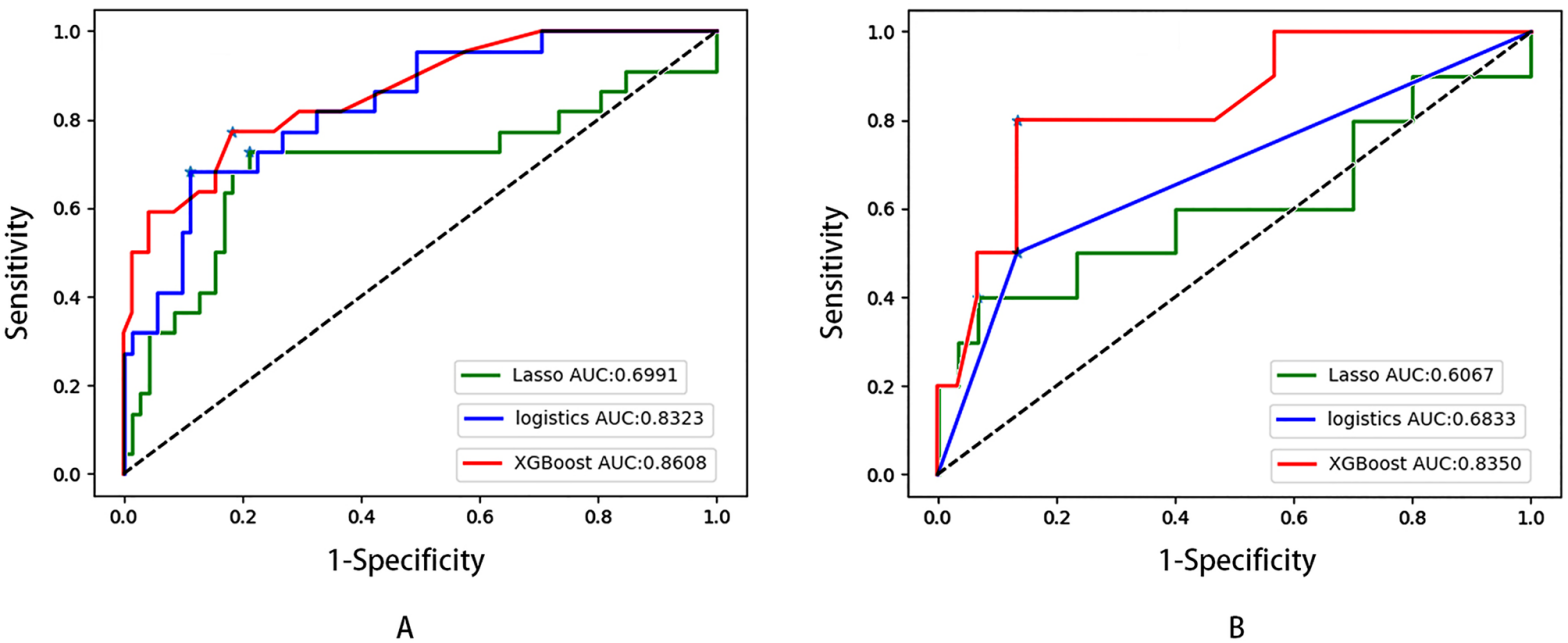
The ROC of the XGBoost algorithm, LASSO regression, and the logistic regression model in the training set (**A**) and the validation set (**B**).

We used the same training group and validation group, the AUC of LASSO regression in both the training and validation sets were lower than that of XGBoost and logistic regression. (Fig. 5A,B) The AUC of the XGBoost model in the training set was 0.861(95%CI 0.792–0.884), which is similar to the AUC of logistic regression (0.832, 95%CI 0.738–0.927, shown in Fig. 5A). As seen in Fig. 5B, the AUC of the logistic model was 0.6833(95%CI 0.520–0.970), which was lower than the AUC of the XGBoost model. (0.835, 95%CI 0.655–0.870).

The three prediction models of PC differed in terms of sensitivity, specificity, accuracy, false positives, and false negatives (shown in Table 4). The XGBoost model, in particular, had 2 false negatives compared to 5 false negatives for the logistic model and 6 false negatives for the LASSO regression in the validation group. The AUC was significant for each algorithm (*p* < 0.05). In the XGBoost model, the optimal threshold (0–1) was similar between the training group and the validation group, with values of 0.455 and 0.456. In the training set, the cut-off value was 0.772 for LASSO and 0.731 for logistic regression, while in the validation set it was 0.807 for LASSO and 0.623 for logistic regression. The multiple logistic and linear regressions with both 6 variables of LASSO regression and 8 variables of the XGBoost model did not observe significant differences in predictive accuracy, sensitivity, and specificity (Table 2 in supplementary). The calibration of the XGBoost model was 1.957 (*p* < 0.05) according to the Hosmer–Lemeshow test, which was higher than the logistic model calibration (*p*-value: 0.465).

**Table 4:** Performance in XGBoost, LASSO regression, and logistic regression of prediction model for PC.

Model	Sensitivity	Specificity	Accuracy	False-positive (FP)	False-negative (FN)
Training					
Logistics	0.682	0.887	0.832	8	7
XGBoost	0.773	0.817	0.861	13	5
LASSO	0.727	0.789	0.774	15	6
Validation					
Logistics	0.500	0.867	0.683	4	5
XGBoost	0.800	0.867	0.835	4	2
LASSO	0.400	0.933	0.800	2	6

The optimal threshold (0–1) was 0.455 for the XGBoost model, 0.772 for LASSO, and 0.731 for logistic regression in the training set, while it was 0.456 for XGBoost model, 0.807 for LASSO, and 0.623 for logistic regression in the validation set.

## Discussion

Cognitive decline is common among patients with PHPT, which is characterized by elevated PTH and serum calcium. Several reports have identified that patients with PHPT appear to have an increased incidence of cognitive dysfunction^40–42^. According to a current systematic review, cognitive impairment in PHPT is more likely to be associated with elevated PTH levels rather than hypercalcemia^13^. However, the mechanism for inducing the impairment of cognition remains to be studied. The details of these relationships between cognitive impairment and serum biomarkers, such as PTH and serum calcium, merit further investigation. Despite the conclusion of the current 5th International Workshop that cognitive evaluation for patients with PHPT is not a necessary test^43^. The cognitive function assessment in patients with parathyroid cancer, who may have cancer-related cognitive impairment, may offer new ideas to distinguish benign parathyroid disease from parathyroid cancer.

High-level PTH may play a role in cognitive dysfunction and cerebrovascular diseases by way of PTH2 receptors (PTHrP) scattered throughout the arteries of the cerebral cortex. PTH2 receptor expression is dominated in limbic, hypothalamic, and sensory areas, particularly hypothalamic periventricular neurons and median eminence nerve terminals^13,41,44–46^. The cerebral area responsible for these functions is the same as the area where PTH receptors are distributed. Therefore, it seems reasonable to speculate that the cognitive decline in patients with PHPT might be proportionally interrelated with PTH level. Bjorkman found that elevated levels of PTH was associated with MMSE in a five-year follow-up in a general-aged population^44^. Unlike these previous reports^41,45–47^, only a weak link between cognition deficit and elevated PTH level was observed in MMSE (Spearman correlation = − 0.172 *p* = 0.048 < 0.05) based on our data, while MOCA failed to show a correlation with PTH level (*p* = 0.474 > 0.05). The reasons for this inconsistency may be as follows. One reason is that we excluded the influence of age, education, depression, and anxiety on cognitive performance by comparing the PC group to the matched control group, which has been neglected in previous research. Another may be that the effect of peripheral cancer on cognitive impairment could not be excluded in parathyroid cancer because patients often experience significant neurocognitive decline, as has been observed in other cancers^14^. Based on the physiological perspective, cognitive decline may be associated with the distribution of PTH2 receptors in different pathological states, which requires to be proved by subsequent experiments. The modification of PTH secretion by serum calcium is changed in patients with PHPT. In accordance with previous studies^13,17^, we found no link between calcium levels and neurocognitive function (MMSE: *p* = 0.106 > 0.05; MOCA: *p* = 0.506 > 0.05). Additionally, a lack of vitamin D could lead to cognitive decline in the older adult^48^. Though the mean concentrations of vitamin D in patients both in PC and BP are lower than normal, we didn’t observe a link between decreased vitamin D and impaired cognition both in MMSE and MOCA (MMSE: *p* = 0.716 > 0.5; MOCA: *p* = 0.834 > 0.5). Further, it needs more mechanistic experiments to determine whether these effects are related to the neurocognitive aspects of PC.

By self-reporting neurocognitive symptoms (presenting difficult concentration and memory problems), Daniel Repplinger reported that neurocognitive dysfunction may be used as a predictor of parathyroid hyperplasia^49^. In our study, we proposed the pre-surgery cognitive function as a potential indicator for PC and both MMSE and MOCA could be used as robust tools for assessing the cognition of patients with PC (*p* < 0.05). In addition, MMSE was superior in detecting cognition in distinguishing patients with PC from PHPT. This is more likely due to MMSE stability of no influence on sex and good internal consistency in measuring the severity of cognitive problems^50–52^. Those deteriorations of cognitive function in patients with PC are primarily characterized by impaired attention, diminished calculative accuracy, difficulties in extracting acquired information from memory, and scathed visual constructive abilities. (Table 5. attention and calculation *p* = 0.003; recall *p* = 0.007; language and visual construction *p* = 0.03). Notably, a similar phenomenon was reported by Janelsins et al.^53^ who found that patients with stage I-IIIC breast cancer have significant cognitive impairment before treatment, particularly in the areas of memory, attention, and executive function. Whether a similar phenomenon is observed in other cancers needs further investigation.

**Table 5:** Distribution of the scores of the MMSE between PC and BP.

Factors	BP group (n = 101)	PC group (n = 32)	P	AUC
Orientation to time and place (0–10)	10 (10, 10)	10 (10, 10)	0.301	0.551
Registration (0–3)	3 (3, 3)	3 (3, 3)	0.072	0.524
Attention and Calculation (0–5)	5 (3, 5)	3 (1, 5)	0.003*	0.677
Recall (0–3)	2 (1, 3)	1 (0, 2)	0.007*	0.71
Language and Visual Construction (0–9)	9 (8, 9)	8 (7, 9)	0.03*	0.66

*p<0.05 for parathyroid carcinoma versus benign parathyroid disease.

Based on the above perspective and the study data, we developed three prediction models for PC on the XGBoost algorithm, LASSO regression, and logistic regression by preoperatively taking scores of MMSE and clinical features into account (in Table 4). As far as we are aware, this is the first time that the use of XGBoost and LASSO regression in the prediction of PC has been presented. The sensitivities of the three models were 0.773, 0.727 and 0.682, and their specificities were 0.817, 0.789, and 0.887, respectively. In comparison to the traditional statistical approach, the XGBoost model could learn complex nonlinear decision boundaries through boosting, whereas linear models such as logistic regression may ignore interactive relationships of the multiple indicators in non-linear and perform the suboptimal outcome^54–56^. In our study, the predictive performance of the XGBoost model, with the lowest false negative rate, was superior to that of the logistic model and LASSO regression model. With a low percentage of underdiagnosis, it would be sensitive to forecast the likelihood of cancer in PHPT avoiding the second surgery. In addition, the XGBoost model can learn the optimal strategy for the best direction to effectively handle missing values in the data by sparsity-aware split-finding. This method enables the XGBoost model to decide on missing data during the training process, which frequently leads to improved model performance. Although the median or mean values from the data are typically used to impute missing values, the many individual differences may still have an impact on the outcomes. In this study, there are currently two PC patients with missing values of ALP and 24-h urinary calcium in the training set. Even after handling missing values, the predictive performance of the XGBoost model remained superior to LASSO and logistic regression. In addition, we processed missing values into the origin validation dataset. Even if the extent of missing values for each variable reached 10%, the predictive performance of the XGBoost remained superior to the Logistic and LASSO regression models, especially in significantly reducing false negative rates (AUC:0.807 vs 0.503 vs 0.513. Table 1 in supplementary). Furthermore, when the extent of missing value increased to 20%, the XGBoost model still outperformed LASSO and Logistic regression with 10% missing data. Thus, whether missing values are present or not, the XGBoost model still demonstrated good predictive performance, indicating its ability to handle missing data effectively while still maintaining high accuracy in prediction. According to three-fold cross-validation, The XGBoost model outperformed Logistic and LASSO regression in terms of accuracy and AUC (accuracy of the model: 0.842 vs 0.773 vs 0.800; AUC: 0.851 vs 0.723 vs 0.666. Table 3 in supplementary) The three-fold cross-validation method emphasized the consistency of the XGBoost model’s performance, further supporting the validity of our findings. This research may offer a reasonably accurate and convenient tool for predicting PC.

Our study has serval limitations. First, the neurocognitive psychological evaluations were subjective assessments that might be influenced by individuals. More PHPT patients need to be included to validate the predictive model. Second, this is considered a preliminary study due to a single-center study with an inevitably small sample size which may affect the generalizability of the findings. Future studies with a larger number of participants in multi-center are required. Third, the model was developed based on our internal verification in the Chinses population, consequently unknowing in other populations. Furthermore, multiple populations need to be used to validate the prediction models developed by XGBoost.

In conclusion, our research demonstrated that the pre-surgery cognitive function might be a potential predictor for PC in patients with PHPT. MMSE is superior to MOCA in evaluating cognition function in PHPT patients and differing PC from BP. Preoperative cognitive assessment of MMSE is necessary for patients with PHPT suspected of PC. The XGBoost model, which had a better performance than the LASSO and logistic model, could predict PC based on pre-surgery cognitive function and clinical features. The performance of the prediction model for PC based on the XGBoost model needs to be further verified in larger populations of PHPT patients.

### Supplementary Information

Supplementary Tables.

## Data Availability

The datasets analyzed during the current study are not available since we are still collecting more data for further study, but are available from the corresponding author on reasonable request.
