# Supplementary material for: Machine learning-based prediction models for parathyroid carcinoma using pre-surgery cognitive function and clinical features
Source: Sci Rep. 2023 Nov 3;13:19007. doi: 10.1038/s41598-023-46294-7 (PMC10624903; doi:10.1038/s41598-023-46294-7)
Supplement: Supplementary file 1 — Supplementary Tables. [file 41598_2023_46294_MOESM1_ESM.docx]

**Supplementary**

**Table 1. The presence of missing values in the validation set affects the predictive performance of the XGBoost model, LASSO regression and Logistic regression**

| Method | Training set | Validation set | AUC | Sensitivity | Specificity | False-positive  （FP） | False-negative  (FN) |
| --- | --- | --- | --- | --- | --- | --- | --- |
| XGBoost model | Origin | 10%Missing^a^ | 0.807 | 0.800 | 0.767 | 7 | 2 |
| Logistic regression | Origin | 10%Missing^a^ | 0.503 | 0.600 | 0.833 | 5 | 4 |
| Lasso regression | Origin | 10%Missing^a^ | 0.513 | 0.250 | 0.929 | 2 | 7 |
| XGBoost model | Origin | 20%Missing^b^ | 0.758 | 0.800 | 0.700 | 9 | 2 |

^a^: The extent of missing values is 10% in validation set, the missing values among the three groups are from the same dataset. b: The extent of missing values is 20% in validation set, based on existing 10% of missing data^a^, another 10% increase in the proportion of missing values.

**Table 2. Logistic regression and multivariable liner regression of 6 variables and 8 variables in prediction model for PC**

| Method | Sensitivity | Specificity | Accuracy | False-positive  （FP） | False-negative  (FN) |
| --- | --- | --- | --- | --- | --- |
| Logistic  6 variables (LASSO-based method for PC prediction) | 0.409 | 0.930 | 0.806 | 5 | 13 |
| 8 variables (XGBoost-based method for PC prediction) | 0.409 | 0.944 | 0.817 | 4 | 13 |
| Multivariable liner regression | |  |  |  |  |
| 6 variables (LASSO-based method for PC prediction)  Y=1.242+0.281*sex+0.215*PTH-0.358*MMSE | 0.727 | 0.789 | 0.699 | 15 | 6 |
| 8 variables (XGBoost-based method for PC prediction)  Y=1.292+0.267*sex+0.166PTH-0.354MMSE | 0.727 | 0.789 | 0.700 | 15 | 6 |

^6 variables: LASSO-based method of PC prediction, including sex, MMSE, PTH, calcium, vitamin D, and phosphorus; 8 variables: the XGBoost-based method for PC prediction, including sex, MMSE, PTH, alkaline phosphatase, calcium, 24-hour urinary calcium, 25-hydroxy vitamin D, and phosphorus.^

**Table 3. Three-ford validation of Logistic regression, XGBoost model, LASSO regression in prediction model for PC**

| Method | | AUC | Sensitivity | Specificity | Accuracy |  |  |
| --- | --- | --- | --- | --- | --- | --- | --- |
| Train 1(training set=group 1+group 2, validation set=group 3) | | | | | |  |  |
| Logistic regression | | 0.610 | 0.455 | 0.912 | 0.800 |  |  |
| LASSO-based method for PC prediction | | 0.754 | 0.818 | 0.676 | 0.717 |  |  |
| XGBoost-based method for PC prediction | | 0.957 | 0.909 | 0.941 | 0.889 |  |  |
|  | Train 2(training set=group 2+group 3, validation set=group 1) | | | | |  |  |
| Logistic regression | | | 0.888 | 0.714 | 0.972 | 0.925 |  |
| LASSO-based method for PC prediction | | | 0.757 | 0.857 | 0.784 | 0.797 |  |
| XGBoost-based method for PC prediction | | | 0.863 | 0.714 | 0.946 | 0.886 |  |
|  | | | Train 3(training set=group 1+group 3, validation set=group 2) | | | | |
| Logistic regression | | | | 0.500 | 0.917 | 0.500 | 0.614 |
| LASSO-based method for PC prediction | | | | 0.657 | 0.643 | 0.867 | 0.806 |
| XGBoost-based method for PC prediction | | | | 0.733 | 0.500 | 0.900 | 0.750 |
| Average of three trainings | | | | | | | |
| Logistic regression | | | | 0.666 | 0.695 | 0.795 | 0.800 |
| LASSO-based method for PC prediction | | | | 0.723 | 0.773 | 0.776 | 0.773 |
| XGBoost-based method for PC prediction | | | | 0.851 | 0.708 | 0.929 | 0.842 |

^we randomly separated all enrolled PHPT patients into three groups, namely Group 1, Group 2, and Group 3. Group 1 consisted of 44 patients (BP=36, PC=8), Group 2 had 44 patients (BP=32, PC=12), and Group 3 included 45 patients (BP=32, PC=13). The baseline data across these three groups did not exhibit significant differences.^
